# Association between wrist-worn actigraphy and the MDS-UPDRS Parkinson’s disease rating scale through machine learning: an exploratory study

**DOI:** 10.3389/fdgth.2026.1876052

**Published:** 2026-07-13

**Authors:** Gent Ymeri, Sara Caramaschi, Alban Haton, Carl Magnus Olsson, Myrthe Wassenburg, Per Svenningsson, Dario Salvi

**Affiliations:** 1 Sustainable Digitalisation Research Centre, Computer Science and Media Technology, Malmö University, Malmö, Sweden; 2 Institut National Polytechnique Clermont-Auvergne, Clermont, France; 3 Clinical Neuroscience, Karolinska Institutet, Stockholm, Sweden; 4 Center for Neurology, Academic Specialist Center Torsplan, Stockholm, Sweden

**Keywords:** actigraphy, GENEActiv, machine learning, MDS-UPDRS, Parkinson’s disease, wrist-worn, wearable sensors, self-supervised learning

## Abstract

**Introduction:**

Parkinson's disease (PD) is typically assessed during short clinical visits using rating scales such as the Movement Disorder Society-Unified Parkinson's Disease Rating Scale (MDS-UPDRS). These assessments provide only a snapshot of symptom severity and may not capture fluctuations in daily life. In this study, we examined whether wrist-worn actigraphy can be used to estimate MDS-UPDRS scores in people with Parkinson's disease (PwP).

**Methods:**

Continuous accelerometer recordings at 25 Hz were collected over up to 28 days using GeneActiv devices. From these recordings, three feature representations were derived: non-embedding actigraphy features, self-supervised accelerometer embeddings, and a combined feature set. A small set of regression models was evaluated using strict leave-one-participant-out cross-validation (LOPO-CV).

**Results:**

Estimation performance varied across targets and feature sets. The strongest result was observed for MDS-UPDRS Part IV, where non-embedding features with Elastic Net achieved a mean absolute error (MAE) of 1.6 and a correlation of 0.83 between estimated and actual values. The combined feature set performed best for Part I (MAE = 3.0, *r* = 0.60), Part III (MAE = 8.2, *r* = 0.47), and the total MDS-UPDRS score (MAE = 13.3, *r* = 0.49), whereas non-embedding features performed best for Part II (MAE = 2.7, *r* = 0.61). Embedding-only models were competitive for some outcomes, but were not the best overall.

**Discussion:**

Overall, the results show that month-long wrist-worn actigraphy contains information related to PD severity in daily life, although estimation accuracy remains limited and depends on the MDS-UPDRS target. Wearable-derived measures may therefore provide complementary information to clinical assessments, particularly for motor complications.

## 1 Introduction

Neurological disorders are a major cause of disability and often lead to declines in physical and mental functioning, thus harming life quality [1]. Among these conditions, Parkinson’s disease (PD) is the second most prevalent neurodegenerative disease [2, 3]. It is characterized by non-motor and motor symptoms that fluctuate during everyday life, with motor symptoms being central to the diagnosis [4]. To date, PD is typically assessed during episodic in-clinic visits, which may not fully capture symptom variability outside the clinical setting. These assessments are also often complemented by retrospective patient-reported diaries and questionnaires, which may be affected by recall bias and may not accurately reflect motor behaviour in free-living conditions [5, 6]. To address these limitations, wearable sensors have emerged as promising tools for continuous and objective monitoring of movement behaviour in people with Parkinson’s (PwP) under real-world conditions [5, 7–10]. In particular, wrist-worn accelerometers can capture long-term actigraphy that reflects motor behavior across days and contexts beyond brief clinical visits [11–13].

Notwithstanding promises, it remains unclear which actigraphy-derived features are most informative for clinically relevant PD severity outcomes and how they relate to established measures of disease severity. Despite the growing availability of wrist accelerometry, it is still uncertain which feature representations provide meaningful information about PD severity and whether learned representations from unsupervised ML could add value beyond established signal processing approaches for capturing physical activity and circadian metrics. More recently, machine learning (ML) and deep learning (DL) approaches have been applied to raw accelerometer signals to improve symptom severity assessment [14, 15]. However, many of these approaches have focused on supervised or more structured settings rather than long-term free-living monitoring. Thus, the relationship between learned representations and established clinical scales such as MDS-UPDRS remains insufficiently explored.

The MDS-UPDRS is widely used to assess the severity of motor and non-motor symptoms in PD and therefore provides a clinically relevant reference outcome. In this work, we examined whether features extracted from month-long GeneActiv recordings were related to MDS-UPDRS severity scores, including GGIR-derived activity summaries, nonparametric circadian rhythm metrics (e.g., inter-daily stability, intra-daily variability, relative amplitude), measures of activity fragmentation, and self-supervised accelerometry embeddings aggregated over time. We hypothesized that greater clinical severity would be associated with lower overall activity, higher fragmentation, and weaker/less regular circadian organization, and that embedding-based representations would capture complementary patterns of motor behavior beyond these features.

## 2 Related works

A number of studies have investigated the use of wearable accelerometers to characterize motor behavior in real-world settings. For example, Adams et al. [7] deployed multiple wearable accelerometers to quantify activity patterns, gait characteristics, and tremor prevalence in PwP during both clinical assessments and free-living monitoring. Their work demonstrated that wearable sensors can capture meaningful differences between PwP and healthy controls in daily walking activity, including reduced step counts and altered gait characteristics in PD. Additionally, signal processing methods enabled quantification of tremor occurrence across daily activities by comparing the most-affected hand with the less-affected hand. For the most-affected hand, tremor was more prevalent in terms of duration during the day, highlighting the potential of wearable sensing to reveal symptom manifestations beyond brief clinical visits. The monitoring duration in this study was limited to approximately two days and relied on multiple body-mounted sensors, which may limit scalability for long-term monitoring.

Other work has focused on using wearable sensor data to model disease progression over time and estimate MDS-UPDRS Part III. Sotirakis et al. [16] collected longitudinal inertial sensor data during standardized walking and postural sway tasks over 18 months. From a large set of extracted kinematic features, the authors identified a subset that exhibited statistically significant temporal changes and used ML models to estimate motor severity scores (MDS-UPDRS Part III). Their results suggested that sensor-derived estimates could detect progression earlier than the clinical MDS-UPDRS Part III score, highlighting the potential of wearable sensing for sensitive monitoring of disease evolution, with their best-performing model achieving a root-mean-square error (RMSE) of 10.02, which is well above the minimum clinically important difference (MCID) reported for Part III in Mishra et al. [17]. In addition, their approach remains largely restricted to structured laboratory tasks and relies on predefined kinematic features extracted from controlled assessments.

Other work has also investigated the use of wearable sensors to automatically estimate clinical severity scores during structured motor tasks. Bremm et al. [18] used a wrist-worn inertial measurement unit (IMU) sensor consisting of accelerometer, gyroscope, and magnetometer to quantify upper-limb motor tasks from the MDS-UPDRS Part III examination, including finger tapping, pronation–supination, and hand opening and closing. Using ML models, trained on extracted time- and frequency-domain features, they demonstrated high (94% accuracy) in classifying movement tasks and achieved moderate performance (68% to 92% AUROC) in classifying corresponding motor clinical scores. These findings illustrate the feasibility of automated scoring of motor assessments; however, such approaches remain dependent on structured task performance and supervised learning frameworks.

Similarly, Zhao et al. [19] investigated how to identify a minimal set of representative activities from the MDS-UPDRS Part III examination for the self-assessment of Parkinson’s disease based on wearables. In their study, movement data were collected using a single wrist-worn inertial sensor containing accelerometers and gyroscopes while participants performed 14 standardized MDS-UPDRS Part III motor tasks under supervised clinical conditions. The tasks included upper-limb and coordination assessments commonly used in neurological examinations, such as hand opening and closing, finger tapping, and pronation–supination movements. From the recorded inertial signals, the authors extracted statistical and frequency-domain features and trained machine learning classifiers to evaluate both PD diagnosis (PD vs. healthy controls) and disease severity classification grouped into three classes (mild: scores 1–2, moderate: score 3, severe: score 4). To determine which activities were most informative, the authors evaluated classification performance for both individual tasks and combinations of tasks, selecting activities that provided complementary information with low inter-task correlation. Gradient boosting–based classifiers were used for estimation, and the results showed that combining a small subset of tasks substantially improved performance compared to single-activity models. The best-performing configuration achieved an F1 Score of 95.75% for PD diagnosis and a fine-grained disease-severity classification accuracy of 82.41%. These findings suggest that a reduced set of representative activities can lower the burden of clinical assessments while maintaining strong estimation performance. However, the severity estimation was based on MDS-UPDRS item scores corresponding to specific structured tasks, rather than overall disease severity, and therefore remains limited. In addition, similarly to other works, this approach relies on structured, supervised clinical tasks and therefore does not capture the variability in motor behavior during free-living daily activities. Recent work has also applied multidimensional wearable movement sensor features to PD detection, showing that ensemble-based ML classifiers, feature ablation, and interpretable SHAP analyses can help identify movement features that distinguish PD from non-PD participants [20].

Despite the growing literature on wearable sensing for PD, a number of gaps remain. Existing work has often focused on PD classification [20], structured task-based assessments conducted in clinical settings or during short-term monitoring periods [21], rather than continuous observation of motor behavior in daily life. Moreover, relatively few studies investigate long-term free-living accelerometry or examine how wearable-derived representations relate to the different aspects of clinical severity measured by scales such as the MDS-UPDRS.

In this work, we address these gaps by investigating the relationship between 1-month wrist actigraphy and clinical outcomes in PwP. Using recordings from GeneActiv devices, we examine multiple features derived from accelerometer data, including activity summaries, nonparametric circadian rhythm metrics, measures of activity fragmentation, and self-supervised embeddings learned from raw accelerometer signals. By evaluating associations between these representations and MDS-UPDRS scores, our study aims to better understand whether there is an association between the different parts of MDS-UPDRS and wrist-wearable actigraphy, and which aspects of long-term motor behavior captured by wearable sensors are most informative for clinical severity.

## 3 Material and methods

### 3.1 Data

This analysis was based on data from the ParkApp study [22], which received ethical approval from the Swedish Ethical Review Authority (application number 2022-02885-01). Thirty individuals diagnosed with PD were enrolled through the Academic Specialist Center Torsplan within Stockholm Health Services, Sweden. Participants underwent clinical evaluation using the MDS-UPDRS at baseline (day 1) and again at the end of the two-month study period (day 60).

Throughout the study, participants wore a GENEActiv wrist-worn accelerometer, which collects raw and unfiltered accelerometer data, positioned on the dominant wrist or, when relevant, on the side most affected by PD symptoms. Prior to deployment, devices were fully charged and programmed to record continuous tri-axial acceleration data at a sampling rate of 25 Hz. Recordings continued until battery depletion, yielding up to maximum 28 days of uninterrupted data per participant. Following data quality assessment, recordings from 27 participants were retained for analysis; exclusions were due to device failure (n=2) and study withdrawal (n=1).

### 3.2 Pre-processing, and feature extraction

Raw triaxial acceleration, ambient light, and skin temperature were recorded using GeneActiv devices, which store the data in binary files. These raw files were converted to comma-separated values (CSV) files with the GENEAread package for R.

Activity and sleep-related metrics from raw accelerometry files were analyzed using the GGIR [23] package in R, which performs automated calibration, non-wear detection, and signal processing directly from raw acceleration signals. GGIR internally aggregates the signal into short epochs (default 5-s windows) and derives time-domain physical activity measures and circadian rhythm indicators. The outputs used were daily summaries of physical activity, sleep parameters, and circadian rhythm metrics, extracted for each participant.

Whereas, circadian rhythm fragmentation metrics were computed using the nparACT [24] package for R. For this purpose, raw acceleration signals were aggregated to a one-minute level by averaging the vector magnitude ($\sqrt{x^{2}+y^{2}+z^{2}}$) and converted to CSV files using the aforementioned GENEAread package. Afterwards, these minute-level activity series were then provided as input to nparACT, which computed nonparametric circadian metrics, including interdaily stability (IS), intradaily variability (IV), relative amplitude (RA), and L5 and M10 activity levels. Other actigraphy summary measures were extracted using the Actigraphy Toolbox [25] following the methods described in Tsanas [26] and Tsanas et al. [27].

**Figure 1:**
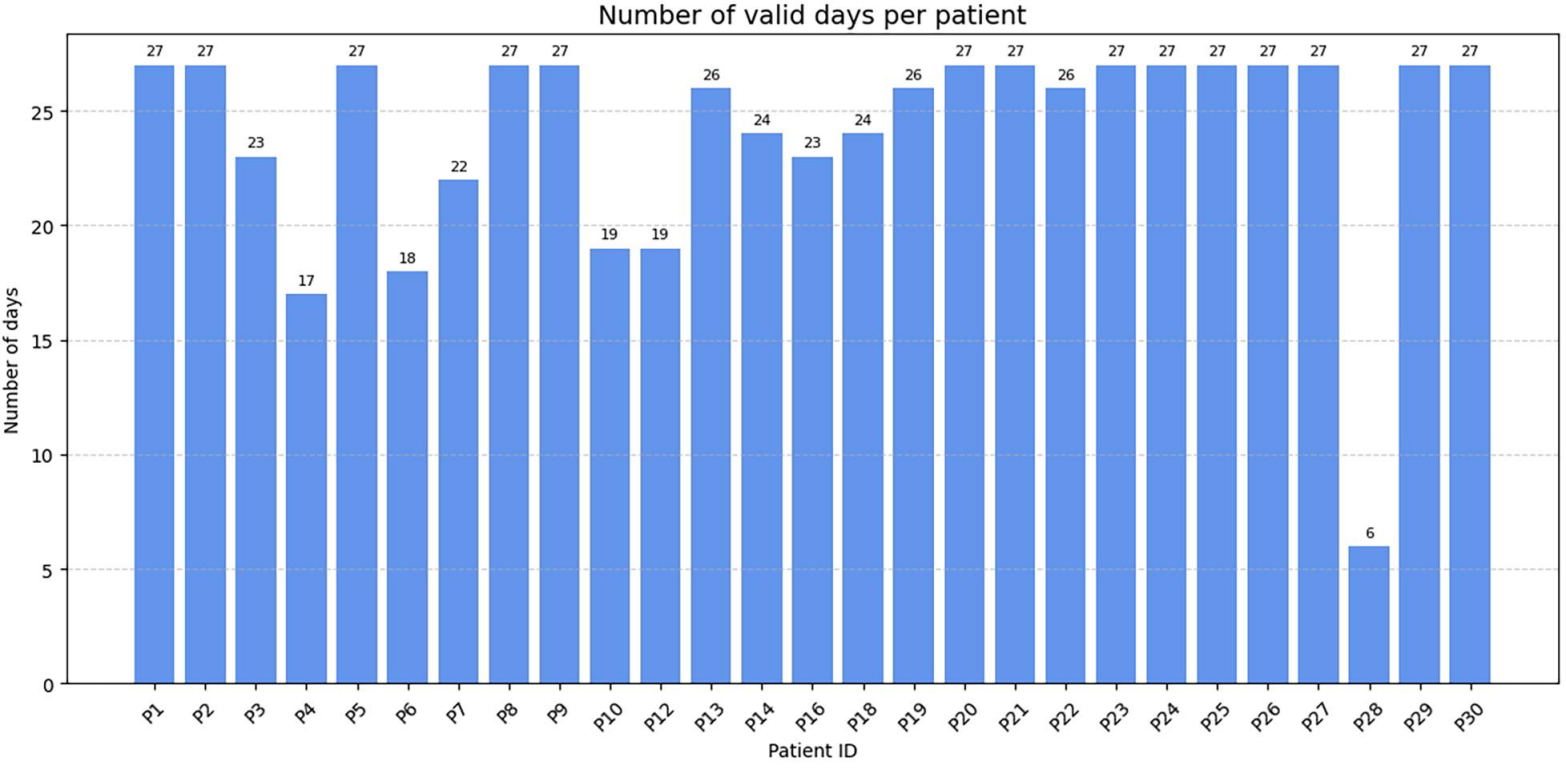
Number of valid days per patient as derived from GGIR package.

Quality-control procedures excluded days with insufficient wear time or incomplete recordings. Only valid days that met the predefined data completeness criteria were retained for analysis, and Figure 1 illustrates the number of valid recording days per participant.

In addition to the above-mentioned activity features, representation learning was applied to derive data-driven features from the raw accelerometer signals. To achieve this, the triaxial acceleration data were resampled to 30 Hz using linear interpolation to match the input requirements of the embedding model. The signals were then segmented into fixed-length windows of 10 s with a 50% overlap (5-s stride). Prior to windowing, the acceleration signals along each axis were standardized per participant using z-score normalization.

Each window was subsequently processed using the pretrained HARNet10 model from the ssl-wearables framework developed by the *OxWearables* [28]. This model is a convolutional neural network trained using self-supervised learning on large-scale wearable accelerometer datasets, 700,000 person days of accelerometer data from the UK Biobank, collected in free-living conditions, to learn generalizable representations of human movement. The output of the model’s feature extraction layer was used to obtain a fixed-length embedding vector for each window.

Finally, window-level embeddings were aggregated at the participant level by computing the mean embedding across all windows, resulting in a single feature vector summarizing the overall movement characteristics of each participant during the monitoring period.

Table 1 summarizes the digital feature groups used in the modelling analysis, their sources, examples of extracted features, and the aggregation level used for model input.

**Table 1:** Overview of digital feature representations used in the machine-learning models.

Feature group	Source/method	Examples of extracted features	Aggregation level used for modelling
Non-embedding features	GGIR	Physical activity summaries, sleep parameters, and circadian rhythm indicators	Daily summaries were averaged across valid recording days to obtain participant-level features.
Non-embedding features	nparACT	Interdaily stability, intradaily variability, relative amplitude, L5 activity, and M10 activity	One-minute activity series were used to compute circadian rhythm metrics, which were then summarized at participant level.
Non-embedding features	Actigraphy Toolbox	Additional actigraphy summary measures, including analogous circadian metrics labelled with the suffix _AT	Features were aggregated at participant level by averaging across recorded valid days.
Embedding features	HARNet10 model from the ssl-wearables framework	Self-supervised accelerometer embedding features extracted from 10-s windows with 50% overlap	Window-level embeddings were averaged across all windows to obtain one participant-level embedding vector.
Combined features	Concatenation of non-embedding and embedding features	Hand-crafted activity/circadian/sleep features combined with HARNet10 embedding features	Participant-level non-embedding and embedding vectors were concatenated before model training.

After all feature sets were extracted, they were then combined into a single dataset comprising GGIR-derived activity summaries, nonparametric circadian rhythm metrics from the nparACT package, actigraphy summary measures from the Actigraphy Toolbox, and self-supervised accelerometer embeddings. To distinguish analogous metrics computed using the different libraries, variables derived using the Actigraphy Toolbox were labeled with the suffix “_AT” (e.g., M10_AT, L5_AT, RA_AT, IS1_AT, IV1_AT). All these features were aggregated at the participant level by averaging across recorded days.

The resulting feature tables were merged using the participant identifier as a key, producing a single participant-level feature matrix. Afterwards, clinical outcome variables were obtained from the baseline MDS-UPDRS assessment and were used as estimation targets in the following modelling analysis. The aim was not to predict future clinical progression, but to estimate baseline MDS-UPDRS severity using the wrist-wearable-derived summaries of habitual behaviour during the monitoring period. A multi-day window was used because daily movement, sleep, and circadian activity patterns can fluctuate from day to day, and a single recording day may not provide a stable representation of functional status. This is particularly relevant for MDS-UPDRS Part IV, which captures time-varying motor complications. The 28-day window was therefore long enough to average over day-to-day variability, but short enough to remain close to the baseline clinical assessment rather than representing longer-term disease progression.

### 3.3 Model training and evaluation

The targets for the model training and evaluation were clinical severity outcomes derived from the baseline MDS-UPDRS assessment, including the Part I, II, III, and IV subscores and the total MDS-UPDRS score (Tot_UPDRS).

To simplify the modelling strategy and avoid information leakage associated with fold-specific feature selection, no additional feature selection was performed. Instead, three feature configurations were evaluated separately:

1. **Non-embedding only:** actigraphy-derived variables from GGIR, nparACT, and the Actigraphy Toolbox;
2. **Embedding only:** the 1024-dimensional self-supervised accelerometer embeddings;
3. **Combined:** non-embedding actigraphy-derived variables together with the self-supervised embeddings.

A small set of regression models suitable for small-sample, high-dimensional data was evaluated, including Ridge regression, Elastic Net, and Partial Least Squares (PLS) regression. For analyses that included embedding features (embedding-only and combined feature sets), an additional PCA+Ridge pipeline was also evaluated. In this approach, principal component analysis (PCA) was applied to the embedding subset within each training fold, while non-embedding features were left unchanged. The embedding dimensions were replaced by a small number of principal components before fitting a Ridge regressor. This dimensionality reduction step was included to mitigate overfitting and improve stability in the high-dimensional embedding space. The number of retained components was fixed to five in the main analysis as a pragmatic low-dimensional representation suitable for the limited sample size. Exploratory sensitivity analyses using 2, 3, 4, 5, 6, and up to 13 components indicated that performance was generally strongest in the low-dimensional range of approximately 4-6 components, whereas larger representations led to poorer results. This supported the use of five retained components in the main analysis. These analyses involved rerunning the complete LOPO-CV workflow with alternative fixed numbers of PCA components. The number of retained components was therefore treated as a model-specification hyperparameter, similar to the other fixed hyperparameters, and was not selected independently within each LOPO-CV iteration. Importantly, PCA itself was not fitted on the full dataset prior to cross-validation. Within each LOPO-CV fold, PCA was fitted only on the training participants and then applied to the held-out participant.

Hyperparameters were fixed for the final reported LOPO-CV analysis, but were not selected through nested cross-validation. Instead, a small exploratory sensitivity analysis was performed separately for each feature configuration by rerunning the complete LOPO-CV workflow under alternative fixed hyperparameter settings. Candidate values were chosen as coarse, conservative values intended to keep model complexity low relative to the small participant-level sample size, rather than as a fine-grained optimization grid. For example, for Ridge models, regularization strengths were explored at broad scales, such as α∈{10,100,1,000}, rather than at adjacent single-unit values. Similarly, PLS was restricted to a small number of latent components, and the PCA component sensitivity analysis was restricted to low-dimensional representations as described above. Because the scale and structure of the estimator space differed substantially across the non-embedding, embedding-only, and combined feature sets, hyperparameters were selected separately within each configuration rather than constrained to be identical across models or feature sets. For the non-embedding feature set, the fixed model configurations were: Ridge regression with α=100, Elastic Net with α=0.5 and $\ell_{1}$ ratio =0.5, and PLS regression with three latent components. For the embedding-only feature set, the fixed configurations were: Ridge regression with α=1,000, Elastic Net with α=0.5 and $\ell_{1}$ ratio =0.5, PCA+Ridge using five principal components with Ridge α=0.5, and PLS regression with two latent components. For the combined feature set, the fixed configurations were: Ridge regression with α=1,000, Elastic Net with α=1 and $\ell_{1}$ ratio =0.9, PCA+Ridge using five principal components with Ridge α=100, and PLS regression with two latent components. These values are not directly comparable across model classes, since Ridge, Elastic Net, and PCA+Ridge impose regularization in different ways and operate on estimator spaces of different dimensionality. Importantly, the exploratory sensitivity analyses changed only the fixed hyperparameter values used in repeated runs of the complete LOPO-CV workflow. Within each LOPO-CV fold, preprocessing parameters, PCA transformations, and model coefficients were estimated using only the training participants, and the held-out participant was used only for evaluation.

Model performance was evaluated using strict leave-one-participant-out cross-validation (LOPO-CV). In each iteration, one participant was held out for testing, and all remaining participants were used for model fitting. All preprocessing steps were performed within the training portion of each fold only. Specifically, when applicable, PCA was fit on the training embedding subset only and then applied to the held-out participant. Standardization using z-score normalization was likewise fit on the training data and applied to the corresponding test participant.

For each feature set, model, and MDS-UPDRS target, held-out estimated scores were pooled across all LOPO-CV folds. Each participant, therefore, contributed one out-of-sample estimate, obtained only when that participant was held out from model training. Thus, MAE, RMSE, and Pearson correlation were then computed once across the full set of observed and held-out estimated scores. The bootstrap confidence intervals were computed by resampling these participant-level observed-estimated score pairs. In addition, model performance was interpreted relative to the Dummy baseline, and the difference in MAE between the Dummy regressor and the best-performing non-Dummy model was used as a simple measure of improvement over baseline.

For the Elastic Net models, we additionally summarized variable-selection frequency across LOPO-CV folds for the interpretable non-embedding feature set. In each fold, after fold-specific standardization and model fitting using only the training participants, features with non-zero Elastic Net coefficients were recorded. For each MDS-UPDRS target, selection frequency was calculated as the number and percentage of LOPO-CV folds in which a feature received a non-zero coefficient. Mean signed and absolute standardized coefficients were also summarized across folds. These frequencies were interpreted descriptively as indicators of feature-selection stability and model contribution, not as definitive feature-importance estimates.

To complement the predictive analyses, we examined participant-level correlations between wearable-derived features and baseline MDS-UPDRS outcomes. Non-embedding features were treated as interpretable actigraphy, sleep, and circadian summaries, whereas self-supervised embedding dimensions were analysed separately as exploratory latent features. Associations were summarized using Spearman correlation coefficients with bootstrap 95% confidence intervals, and false-discovery-rate correction was applied within each MDS-UPDRS outcome using the Benjamini-Hochberg procedure.

#### 3.3.1 Surrogate testing

As a baseline, a Dummy regressor was evaluated in each LOPO-CV fold. This model estimates the mean target value of the training participants and therefore does not use any feature information. It served as a reference for assessing whether the evaluated models provided estimation value beyond a trivial mean-based estimator.

## 4 Results

Estimation performance varied across clinical targets, feature sets, and regression models, and no single approach consistently performed best across all outcomes. The model performance results for each MDS-UPDRS target and feature set are summarized in Table 2.

**Table 2:** Estimation performance across all feature sets under leave-one-participant-out cross-validation.

Feature set	Target	Model	MAE ± 95% CI	RMSE ± 95% CI	r± 95% CI
Non-embedding only					
	Part I	Ridge	**3.79 ± 0.94**	4.50 ± 1.04	0.32 ± 0.36
	Part I	PLS	3.93 ± 0.99	4.71 ± 1.01	0.35 ± 0.32
	Part I	Elastic Net	4.05 ± 0.93	4.73 ± 1.01	0.32 ± 0.32
	Part I	Dummy	4.08 ± 1.03	4.88 ± 0.90	NA
	Part II	Elastic Net	**2.67 ± 0.87**	3.53 ± 1.01	0.61 ± 0.38
	Part II	Ridge	3.14 ± 0.94	4.02 ± 0.93	0.38 ± 0.47
	Part II	PLS	3.25 ± 0.96	4.15 ± 1.18	0.46 ± 0.47
	Part II	Dummy	3.62 ± 1.00	4.49 ± 1.18	NA
	Part III	Ridge	**8.80 ± 2.40**	10.82 ± 2.91	0.23 ± 0.40
	Part III	Elastic Net	9.07 ± 2.57	11.35 ± 3.12	0.41 ± 0.33
	Part III	Dummy	9.42 ± 2.11	10.95 ± 2.01	NA
	Part III	PLS	9.82 ± 2.73	12.22 ± 3.39	0.28 ± 0.40
	Part IV	Elastic Net	**1.55 ± 0.48**	1.99 ± 0.50	0.83 ± 0.12
	Part IV	PLS	1.66 ± 0.49	2.10 ± 0.50	0.81 ± 0.12
	Part IV	Ridge	1.75 ± 0.48	2.17 ± 0.52	0.80 ± 0.15
	Part IV	Dummy	3.12 ± 0.74	3.70 ± 0.88	NA
	Total	Ridge	**13.84 ± 3.95**	17.44 ± 4.45	0.28 ± 0.44
	Total	Elastic Net	13.87 ± 4.13	17.60 ± 4.44	0.49 ± 0.32
	Total	PLS	14.40 ± 4.27	18.39 ± 5.20	0.36 ± 0.44
	Total	Dummy	15.22 ± 4.03	18.67 ± 4.24	NA
Embedding only					
	Part I	PLS	**3.04 ± 0.94**	3.90 ± 1.04	0.58 ± 0.27
	Part I	PCA+Ridge	3.27 ± 0.98	4.15 ± 1.05	0.49 ± 0.32
	Part I	Ridge	3.33 ± 0.97	4.20 ± 0.87	0.45 ± 0.28
	Part I	Dummy	4.08 ± 1.03	4.88 ± 0.90	NA
	Part I	Elastic Net	4.08 ± 1.07	4.98 ± 1.08	0.25 ± 0.31
	Part II	PCA+Ridge	**3.31 ± 0.98**	4.17 ± 1.17	0.31 ± 0.34
	Part II	Ridge	3.54 ± 0.97	4.39 ± 1.15	0.12 ± 0.30
	Part II	Dummy	3.62 ± 1.00	4.49 ± 1.18	NA
	Part II	PLS	3.72 ± 0.99	4.53 ± 1.15	0.20 ± 0.31
	Part II	Elastic Net	4.02 ± 1.25	5.20 ± 1.50	-0.02 ± 0.36
	Part III	Elastic Net	**9.32 ± 2.19**	10.97 ± 2.38	0.39 ± 0.36
	Part III	PCA+Ridge	**9.32 ± 2.04**	10.73 ± 2.06	0.23 ± 0.42
	Part III	Dummy	9.42 ± 2.11	10.95 ± 2.01	NA
	Part III	Ridge	9.42 ± 2.07	10.83 ± 2.36	0.23 ± 0.43
	Part III	PLS	11.24 ± 2.63	13.14 ± 3.19	0.14 ± 0.47
	Part IV	Ridge	**2.43 ± 0.78**	3.20 ± 1.15	0.50 ± 0.43
	Part IV	PLS	2.44 ± 0.75	3.15 ± 1.01	0.55 ± 0.37
	Part IV	PCA+Ridge	2.58 ± 0.83	3.39 ± 1.21	0.48 ± 0.40
	Part IV	Elastic Net	2.62 ± 0.90	3.55 ± 1.44	0.44 ± 0.46
	Part IV	Dummy	3.12 ± 0.74	3.70 ± 0.88	NA
	Total	PCA+Ridge	**14.39 ± 3.88**	17.68 ± 3.94	0.27 ± 0.44
	Total	Ridge	15.14 ± 3.91	18.28 ± 3.70	0.15 ± 0.38
	Total	PLS	15.21 ± 3.90	18.33 ± 4.08	0.28 ± 0.41
	Total	Dummy	15.22 ± 4.03	18.67 ± 4.24	NA
	Total	Elastic Net	16.10 ± 3.36	18.38 ± 3.10	0.31 ± 0.27
Combined					
	Part I	PLS	**3.02 ± 0.88**	3.80 ± 0.92	0.60 ± 0.25
	Part I	Ridge	3.29 ± 0.92	4.10 ± 0.83	0.49 ± 0.27
	Part I	PCA+Ridge	3.65 ± 0.89	4.32 ± 0.98	0.40 ± 0.33
	Part I	Dummy	4.08 ± 1.03	4.88 ± 0.90	NA
	Part I	Elastic Net	4.20 ± 1.05	5.05 ± 1.05	0.10 ± 0.35
	Part II	PCA+Ridge	**3.10 ± 0.90**	3.93 ± 0.87	0.42 ± 0.43
	Part II	Ridge	3.48 ± 0.94	4.30 ± 1.05	0.19 ± 0.33
	Part II	Dummy	3.62 ± 1.00	4.49 ± 1.18	NA
	Part II	Elastic Net	3.63 ± 0.98	4.47 ± 1.04	0.12 ± 0.35
	Part II	PLS	3.69 ± 0.93	4.43 ± 1.06	0.24 ± 0.30
	Part III	Elastic Net	**8.21 ± 2.10**	9.93 ± 2.49	0.47 ± 0.32
	Part III	PCA+Ridge	8.89 ± 2.32	10.80 ± 2.73	0.23 ± 0.38
	Part III	Ridge	9.29 ± 1.97	10.59 ± 2.33	0.26 ± 0.39
	Part III	Dummy	9.42 ± 2.11	10.95 ± 2.01	NA
	Part III	PLS	10.23 ± 2.64	12.31 ± 3.32	0.20 ± 0.44
	Part IV	PCA+Ridge	**1.76 ± 0.48**	2.18 ± 0.51	0.80 ± 0.14
	Part IV	Ridge	2.26 ± 0.74	3.00 ± 1.04	0.57 ± 0.40
	Part IV	PLS	2.33 ± 0.72	3.02 ± 0.98	0.58 ± 0.36
	Part IV	Elastic Net	2.57 ± 0.69	3.16 ± 0.91	0.47 ± 0.42
	Part IV	Dummy	3.12 ± 0.74	3.70 ± 0.88	NA
	Total	Elastic Net	**13.29 ± 3.58**	16.24 ± 3.51	0.49 ± 0.36
	Total	PCA+Ridge	13.81 ± 3.83	17.15 ± 4.03	0.32 ± 0.40
	Total	Ridge	15.05 ± 3.76	18.04 ± 3.45	0.18 ± 0.36
	Total	Dummy	15.22 ± 4.03	18.67 ± 4.24	NA
	Total	PLS	15.36 ± 3.80	18.32 ± 3.90	0.27 ± 0.38

Values are reported as point estimate ± half-width of the bootstrap 95% confidence interval. Within each feature-set block and target, the lowest MAE is shown in bold.

For MDS-UPDRS Part I, the lowest error was obtained with the combined feature set using PLS regression (MAE = 3.0, RMSE = 3.8, r=0.60), representing a modest improvement over the Dummy baseline (MAE = 4.1). For Part II, the best performance was achieved using non-embedding features with Elastic Net (MAE = 2.7, RMSE = 3.5, r=0.61), compared with MAE = 3.6 for the Dummy model. For Part III, the lowest error was observed with the combined feature set using Elastic Net (MAE = 8.2, RMSE = 9.9, r=0.47), but gains over the Dummy baseline (MAE = 9.4) remained limited. For Part IV, the strongest result was obtained using non-embedding features, where Elastic Net achieved the lowest error and highest correlation overall (MAE = 1.6, RMSE = 2.0, r=0.83), thus, outperforming the Dummy baseline (MAE = 3.1). For the total MDS-UPDRS score, the combined feature set with Elastic Net had the best performance (MAE = 13.3, RMSE = 16.2, r=0.49), compared with 15.2 for the Dummy model.

Across feature sets, non-embedding actigraphy-derived variables were most informative for Part II and Part IV, whereas the combined feature set performed best for Part I, Part III, and the total MDS-UPDRS score. Embedding-only models were competitive for some outcomes, particularly Part I (PLS: MAE = 3.0, r=0.58), but did not outperform the best models from the other feature sets. Overall, all best-performing models showed improvement over the Dummy baseline, although the magnitude of this improvement varied across targets, with the clearest gain observed for Part IV, which also showed the strongest correlation (r=0.83).

**Figure 2:**
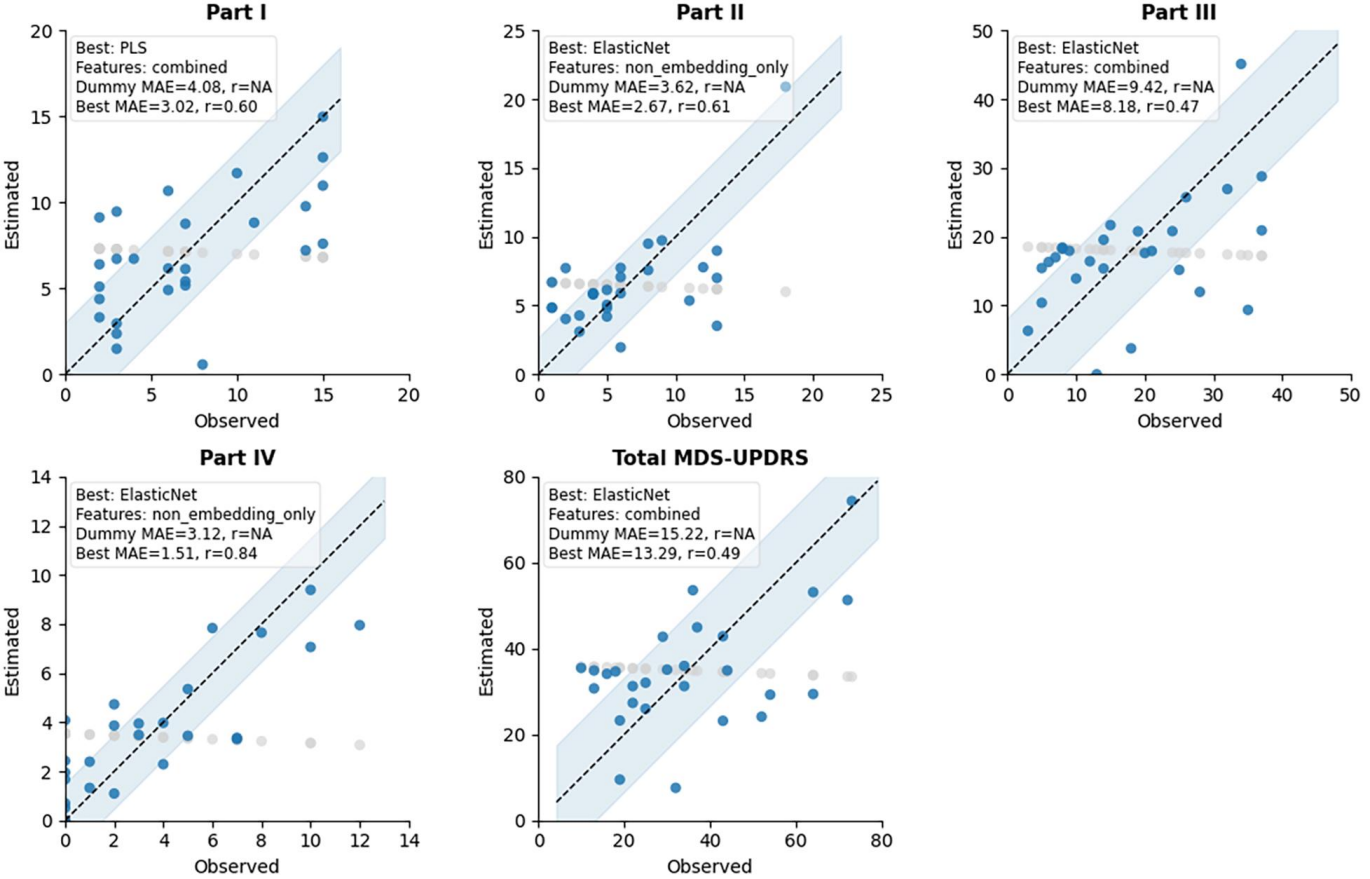
Observed vs. estimated MDS-UPDRS outcomes for the dummy baseline (grey) and the best-performing non-dummy model (blue) for each target. The dashed diagonal indicates perfect agreement (y=x). The shaded blue band represents ±1 MAE around the identity line for the best-performing model. For each outcome, the annotation box reports the selected best model, feature set, MAE, and Pearson correlation. Pearson correlation is shown as NA for the dummy baseline because, under leave-one-participant-out cross-validation, its fold-specific mean estimations yield a correlation that is not meaningfully interpretable.

These results can also be seen in Figure 2, which illustrates the relationship between observed and estimated scores for the Dummy baseline and the best-performing model for each target. The best models generally followed the overall trend in the observed data more closely than the baseline, although estimations remained somewhat compressed toward the mean for some outcomes, particularly for Part III and the total MDS-UPDRS score. Also visually, the strongest agreement between observed and estimated values was observed for Part IV, whereas performance for Parts I-III was more modest.

As a supplementary descriptive analysis, we summarized Elastic Net feature-selection frequency across LOPO-CV folds for the non-embedding feature set. The top retained features for each MDS-UPDRS target are reported in Supplementary Table S3, ranked by selection frequency and mean absolute coefficient. A number of activity, sleep, and circadian rhythm features were retained across all 27 LOPO-CV folds, and for brevity, only the top 10 features per MDS-UPDRS target are shown.

Exploratory participant-level association analyses showed that FDR-significant associations were limited to MDS-UPDRS Part IV. For non-embedding features, these associations primarily involved activity intensity, activity duration, and activity-bout measures. In the separate embedding-dimension analysis, 52 embedding dimensions were associated with Part IV after FDR correction, while no embedding dimensions survived correction for the other outcomes. The full association results are reported in Supplementary Tables S1 and S2.

## 5 Discussion

This study investigated the feasibility of estimating MDS-UPDRS severity scores from wrist-worn actigraphy in PwP. Using up to 28 days of continuous GeneActiv recordings, we evaluated whether free-living movement patterns could provide information related to the MDS-UPDRS Part I-IV subscores and the total MDS-UPDRS score. Considering the limited amount of PwP we needed to limit the risk of overfitting. Thus, the final analysis used a small set of fixed regression models under strict LOPO-CV, and compared three feature configurations separately: non-embedding actigraphy-derived features, self-supervised accelerometer embeddings, and their combination.

Overall, the results indicate that long-term wrist actigraphy contains some information related to PD severity, although the estimation value varied across targets. The clearest and most consistent finding was observed for MDS-UPDRS Part IV, for which the strongest performance was obtained using non-embedding actigraphy-derived features. In particular, Elastic Net achieved a MAE of 1.6 and a correlation of 0.83, substantially improving from the Dummy baseline. This can be clinically reasonable, as Part IV reflects motor fluctuations and dyskinesias, which are inherently temporal phenomena and may therefore be more directly expressed in long-term activity patterns captured by wearable sensors in daily life.

The comparison across feature sets suggested that different representations captured different aspects of disease severity. Non-embedding actigraphy-derived variables were most informative for Part II and Part IV, whereas the combined feature set performed best for Part I, Part III, and the total MDS-UPDRS score. Embedding-only models were competitive for some outcomes, particularly Part I, but did not outperform the best models from the other feature sets. This pattern suggests that extracted actigraphy features and learned representations from HarNet10 are not interchangeable, but instead capture different aspects of the behavioural signal.

In contrast to earlier expectations, combining embeddings with non-embedding features provided some benefit for selected targets, particularly Part I, Part III, and the total MDS-UPDRS score. However, these improvements were not consistent across all outcomes, and gains were generally small. This likely reflects the difficulty of feature fusion in a small-sample, high-dimensional setting, where additional estimators can introduce redundancy without substantially improving generalization. Thus, the results suggest that the usefulness of wearable-derived features depends on the target outcome and that combining representations may be beneficial in some targets but not all.

For the embedding-only analyses, PCA-based dimensionality reduction proved to be beneficial only when the representation was strongly compressed. Exploratory sensitivity analyses indicated that performance was generally strongest when the embeddings were reduced to a small number of principal components (4–6), whereas retaining a larger number of components led to poorer results. This suggests that the most informative embedding-based signal is concentrated in a relatively low-dimensional latent space, and that retaining more components may primarily introduce redundant or noisy variation in the present small-sample setting.

Compared with previous studies, the present work differs in that it focuses on passive, free-living monitoring rather than structured clinical tasks. Much of the existing literature has used wearable sensors to analyse standardized motor tasks performed under supervision, such as finger tapping, pronation-supination, gait, or balance assessments [16, 18, 19]. Such approaches can achieve high performance for task-specific scoring, but they rely on controlled conditions and short observation periods. In contrast, the present study examined whether clinically relevant information could be extracted from continuous wrist actigraphy data collected in daily life over a numbre of weeks. This design better reflects natural behaviour, but it also introduces variability unrelated to disease severity, making the estimation task more challenging.

A number of limitations should be considered when interpreting these findings. First, the sample size was small, with only 27 participants retained for analysis, which limits statistical stability and generalizability. Second, although strict LOPO-CV was used and preprocessing was performed within each training fold only, the model settings were intentionally conservative and not optimized using nested cross-validation. This reduced the risk of overfitting, but may also have limited better performance. The fixed hyperparameter settings should therefore be considered indicative. Although preprocessing and model fitting were performed strictly within each LOPO-CV fold, the final hyperparameter choices were informed by exploratory, non-nested sensitivity analyses on the available cohort. These choices were based on coarse, conservative candidate values rather than fine-grained optimization, and local sensitivity checks indicated that small perturbations of continuous regularization parameters produced only minor changes in MAE. Future studies with larger cohorts could further refine these settings using nested cross-validation with a larger cohort. As an additional sensitivity analysis, we repeated participant-level 5-fold cross-validation across 100 random fold assignments. This analysis held out approximately 4-6 participants per fold, rather than one participant at a time. Compared with the LOPO-CV results, the best non-embedding MAE changed from 3.8 to 3.9 for Part I, 2.7 to 3.1 for Part II, 8.8 to 9.0 for Part III, 1.6 to 1.7 for Part IV, and 13.8 to 14.5 for the total MDS-UPDRS score. Thus, estimation error increased only slightly when multiple participants were held out per fold, suggesting that the observed performance pattern was not driven by the LOPO-CV scheme. Third, passive wrist actigraphy primarily captures broad movement behaviour and may not fully represent specific clinical constructs assessed in the MDS-UPDRS, such as rigidity, fine motor impairment, or tremor characteristics. This is likely one reason why some subscales showed only modest improvements over the Dummy baseline.

The interpretation of performance relative to the Dummy baseline is also important. While some targets showed clear improvements, others showed only modest gains, indicating that the wearable-derived signal was not uniformly strong across all MDS-UPDRS parts. In particular, improvements for Part I and Part III were limited despite the use of combined feature representations. These results suggest that long-term actigraphy may provide complementary information for selected aspects of disease severity, rather than a complete proxy for clinical scoring.

When interpreted relative to published minimal clinically important difference (MCID) thresholds for the MDS-UPDRS [17], the present results should be considered cautiously. Among the evaluated outcomes, the lowest estimation errors were observed for Part IV, which also showed the clearest improvement over the Dummy baseline. However, even for this outcome, the best MAE (1.6) remained above the reported MCID range of approximately 0.8-0.9 points. For Part II, the best MAE approached the published MCID threshold, but this was not accompanied by a comparably strong correlation, suggesting limited robustness of the association. Parts I, III, and the total MDS-UPDRS score also remained above their corresponding published MCID thresholds. Overall, these comparisons suggest that the wearable-derived features contain relevant information for selected parts of MDS-UPDRS, but the current models do not yet achieve an error level that would support equivalence with established clinical scoring. Importantly, this comparison should be interpreted as a rough benchmark rather than a formal agreement analysis, since MCID reflects clinically meaningful change in score, whereas MAE reflects estimation error.

Future work should evaluate these findings in larger cohorts and explore whether multimodal digital phenotyping could improve estimation accuracy. In particular, combining passive wrist actigraphy with other sensor modalities or smartphone-based active assessments, as mentioned in Ymeri et al. [22], may provide more complete coverage of the symptom dimensions represented in the MDS-UPDRS. In addition, repeated clinical MDS-UPDRS assessments collected more often and closer in time to the wearable recordings would allow a more direct comparison between passively captured behaviour and symptom severity.

Despite these limitations, the present study shows that wrist-worn actigraphy can contain measurable information related to PD severity in daily life, particularly motor fluctuations. The usefulness of this information appears to be target-dependent, with the strongest signal observed for MDS-UPDRS Part IV.

## 6 Conclusion

Overall, this study shows that month-long wrist-worn actigraphy captures aspects of PD-related motor behaviour in daily life, but estimation accuracy remains limited and varies across clinical targets. The strongest signal was observed for motor complications (Part IV of MDS-UPDRS), indicating that wearable sensors may offer complementary information to clinical assessments, particularly for monitoring symptoms outside the clinic.

## Data Availability

The raw participant-level data are not currently publicly available due to ethical and data protection restrictions. An anonymized/de-identified version may be made publicly available where permissible. Requests should be directed to the corresponding author.
