# Supplementary material for: Association between wrist-worn actigraphy and the MDS-UPDRS Parkinson’s disease rating scale through machine learning: an exploratory study
Source: Front Digit Health. 2026 Jul 13;8:1876052. doi: 10.3389/fdgth.2026.1876052 (PMC13402524; doi:10.3389/fdgth.2026.1876052)
Supplement: Supplementary file 2 [file Supplementaryfile2.pdf]

Table 3 Participant-level associations between non-embedding wearable features and baseline MDS-UPDRS outcomes. Associations are reported as Spearman correlation coefficients with bootstrap 95% confidence intervals, raw p-values, and false-discovery-rate (FDR) adjusted p-values. The table includes only associations with FDR-adjusted $p < 0.05$ within each MDS-UPDRS outcome.

Feature	MDS-UPDRS outcome	Spearman ρ (95% CI)	Raw p	FDR-adjusted p
Nblocks_day_LIG_unbt	Tot4	0.76 (0.51, 0.89)	< 0.001	< 0.001
Nblocks_day_IN_unbt	Tot4	0.75 (0.48, 0.89)	< 0.001	< 0.001
mean_ENMO_mg_0-24hr	Tot4	0.75 (0.49, 0.89)	< 0.001	< 0.001
ACC_day_spt_mg	Tot4	0.75 (0.49, 0.89)	< 0.001	< 0.001
dur_day_LIG_unbt_min	Tot4	0.74 (0.47, 0.88)	< 0.001	< 0.001
Nblocks_day_MOD_unbt	Tot4	0.72 (0.42, 0.87)	< 0.001	< 0.001
dur_day_MOD_unbt_min	Tot4	0.71 (0.41, 0.87)	< 0.001	< 0.001
Nblocks_day_total_LIG	Tot4	0.71 (0.40, 0.87)	< 0.001	< 0.001
Nblocks_day_total_IN	Tot4	0.70 (0.42, 0.86)	< 0.001	< 0.001
ACC_day_mg	Tot4	0.69 (0.41, 0.85)	< 0.001	< 0.001
dur_day_IN_unbt_min	Tot4	0.69 (0.41, 0.86)	< 0.001	< 0.001
Nblocks_day_total_MOD	Tot4	0.69 (0.37, 0.87)	< 0.001	< 0.001
M10	Tot4	0.69 (0.38, 0.87)	< 0.001	< 0.001
mean_ENMO_mg_12-18hr	Tot4	0.69 (0.39, 0.86)	< 0.001	< 0.001
dur_day_total_LIG_min	Tot4	0.68 (0.40, 0.84)	< 0.001	< 0.001
mean_ENMO_mg_18-24hr	Tot4	0.66 (0.33, 0.86)	< 0.001	0.002
Nbouts_day_MVPA_bts_1_5	Tot4	0.63 (0.28, 0.84)	< 0.001	0.004
Nblocks_day_MVPA_bts_1_5	Tot4	0.63 (0.28, 0.84)	< 0.001	0.004
M5_ENMO_mg_0-24hr	Tot4	0.62 (0.30, 0.81)	< 0.001	0.005
MVPA_E5S_T100_ENMO_0-24hr	Tot4	0.61 (0.26, 0.82)	< 0.001	0.005
dur_day_MVPA_bts_1_5_min	Tot4	0.61 (0.26, 0.82)	< 0.001	0.005
Sedentary (min)_AT	Tot4	-0.60 (-0.80, -0.29)	< 0.001	0.006
MVPA_E5S_T100_ENMO_12-18hr	Tot4	0.60 (0.26, 0.83)	0.001	0.006
MVPA_E1M_T100_ENMO_12-18hr	Tot4	0.60 (0.26, 0.83)	0.001	0.006
Nblocks_day_VIG_unbt	Tot4	0.59 (0.29, 0.80)	0.001	0.007
quantile_mostactive60min_mg	Tot4	0.59 (0.24, 0.81)	0.001	0.007
MVPA_E1M_T100_ENMO_0-24hr	Tot4	0.58 (0.24, 0.80)	0.001	0.008
ACC_day_total_IN_mg	Tot4	0.58 (0.27, 0.76)	0.002	0.008
MVPA_E5S_T100_ENMO_18-24hr	Tot4	0.58 (0.22, 0.82)	0.002	0.008
MVPA_E1M_T100_ENMO_18-24hr	Tot4	0.57 (0.23, 0.81)	0.002	0.009
MVPA_E5M_T100_ENMO_0-24hr	Tot4	0.57 (0.23, 0.81)	0.002	0.009
quantile_mostactive30min_mg	Tot4	0.57 (0.22, 0.80)	0.002	0.009
dur_day_VIG_unbt_min	Tot4	0.57 (0.26, 0.79)	0.002	0.009
dur_day_total_MOD_min	Tot4	0.56 (0.20, 0.80)	0.002	0.010
Nblocks_day_total_VIG	Tot4	0.55 (0.20, 0.79)	0.003	0.012
dur_day_total_VIG_min	Tot4	0.55 (0.19, 0.79)	0.003	0.013
MVPA_E5M_T100_ENMO_12-18hr	Tot4	0.54 (0.17, 0.80)	0.003	0.013
mean_ENMO_mg_6-12hr	Tot4	0.54 (0.20, 0.78)	0.004	0.014
Moderate (min)_AT	Tot4	0.53 (0.17, 0.79)	0.004	0.015
MVPA_E5M_T100_ENMO_18-24hr	Tot4	0.53 (0.18, 0.79)	0.004	0.015
Nbouts_day_IN_bts_10_20	Tot4	0.53 (0.17, 0.76)	0.005	0.017
Nblocks_day_IN_bts_10_20	Tot4	0.53 (0.17, 0.76)	0.005	0.017
dur_day_IN_bts_30_min	Tot4	-0.51 (-0.76, -0.15)	0.006	0.021
dur_day_IN_bts_20_30_min	Tot4	0.51 (0.16, 0.73)	0.007	0.023
M10_AT	Tot4	0.51 (0.13, 0.78)	0.007	0.023

Continued on next page

Table 3 Participant-level associations between non-embedding wearable features and baseline MDS-UPDRS outcomes. Associations are reported as Spearman correlation coefficients with bootstrap 95% confidence intervals, raw p-values, and false-discovery-rate (FDR) adjusted p-values. The table includes only associations with FDR-adjusted $p < 0.05$ within each MDS-UPDRS outcome.

Feature	MDS-UPDRS outcome	Spearman ρ (95% CI)	Raw p	FDR-adjusted p
ACC_day_total_LIG_mg	Tot4	0.50 (0.13, 0.75)	0.007	0.024
Nbouts_day_LIG_bts_1_5	Tot4	0.50 (0.17, 0.72)	0.008	0.025
Nblocks_day_LIG_bts_1_5	Tot4	0.50 (0.17, 0.72)	0.008	0.025
dur_day_LIG_bts_1_5_min	Tot4	0.49 (0.16, 0.71)	0.010	0.030
MVPA_E5S_T100_ENMO_0-6hr	Tot4	0.48 (0.15, 0.73)	0.010	0.030
dur_day_IN_bts_10_20_min	Tot4	0.48 (0.10, 0.73)	0.011	0.031
MVPA_E5S_T100_ENMO_6-12hr	Tot4	0.47 (0.09, 0.75)	0.014	0.039
Nbouts_day_IN_bts_20_30	Tot4	0.46 (0.08, 0.72)	0.015	0.040
Nblocks_day_IN_bts_20_30	Tot4	0.46 (0.08, 0.72)	0.015	0.040
MVPA_E5S_B1M80%_T100_ENMO_0-24hr	Tot4	0.46 (0.08, 0.74)	0.015	0.041
MVPA_E1M_T100_ENMO_0-6hr	Tot4	0.46 (0.12, 0.73)	0.017	0.044
MVPA_E5S_B1M80%_T100_ENMO_18-24hr	Tot4	0.45 (0.07, 0.75)	0.018	0.047
ACC_spt_wake_MOD_mg	Tot4	0.45 (0.13, 0.68)	0.019	0.048
MVPA_E5S_B1M80%_T100_ENMO_12-18hr	Tot4	0.45 (0.06, 0.73)	0.019	0.048

Table 4 Participant-level associations between self-supervised embedding dimensions and baseline MDS-UPDRS outcomes. Associations are reported as Spearman correlation coefficients with bootstrap 95% confidence intervals, raw p-values, and false-discovery-rate (FDR) adjusted p-values. The table includes only associations with FDR-adjusted $p < 0.05$ within each MDS-UPDRS outcome. Embedding dimensions are latent model-derived features and are not directly clinically interpretable.

Embedding dimension	MDS-UPDRS outcome	Spearman ρ (95% CI)	Raw p	FDR-adjusted p
embedding_dim843	Tot4	0.71 (0.42, 0.86)	< 0.001	0.017
embedding_dim103	Tot4	0.70 (0.46, 0.86)	< 0.001	0.017
embedding_dim415	Tot4	-0.70 (-0.86, -0.39)	< 0.001	0.017
embedding_dim338	Tot4	0.69 (0.29, 0.92)	< 0.001	0.021
embedding_dim312	Tot4	0.68 (0.36, 0.86)	< 0.001	0.023
embedding_dim632	Tot4	0.67 (0.36, 0.88)	< 0.001	0.024
embedding_dim609	Tot4	0.66 (0.40, 0.83)	< 0.001	0.024
embedding_dim990	Tot4	-0.66 (-0.87, -0.31)	< 0.001	0.024
embedding_dim324	Tot4	0.65 (0.28, 0.88)	< 0.001	0.024
embedding_dim92	Tot4	0.65 (0.36, 0.82)	< 0.001	0.024
embedding_dim433	Tot4	0.65 (0.33, 0.82)	< 0.001	0.024
embedding_dim1000	Tot4	0.64 (0.24, 0.90)	< 0.001	0.025
embedding_dim709	Tot4	0.63 (0.33, 0.82)	< 0.001	0.030
embedding_dim32	Tot4	-0.63 (-0.86, -0.30)	< 0.001	0.033
embedding_dim211	Tot4	0.62 (0.29, 0.84)	< 0.001	0.034
embedding_dim359	Tot4	0.62 (0.35, 0.78)	< 0.001	0.036
embedding_dim797	Tot4	0.62 (0.20, 0.87)	< 0.001	0.036
embedding_dim577	Tot4	0.61 (0.28, 0.81)	< 0.001	0.038
embedding_dim467	Tot4	0.61 (0.26, 0.81)	< 0.001	0.038
embedding_dim222	Tot4	0.61 (0.23, 0.81)	< 0.001	0.039

Continued on next page

Table 4 Participant-level associations between self-supervised embedding dimensions and baseline MDS-UPDRS outcomes. Associations are reported as Spearman correlation coefficients with bootstrap 95% confidence intervals, raw p-values, and false-discovery-rate (FDR) adjusted p-values. The table includes only associations with FDR-adjusted $p < 0.05$ within each MDS-UPDRS outcome. Embedding dimensions are latent model-derived features and are not directly clinically interpretable.

Embedding dimension	MDS-UPDRS outcome	Spearman ρ (95% CI)	Raw p	FDR-adjusted p
embedding_dim977	Tot4	0.61 (0.24, 0.83)	< 0.001	0.039
embedding_dim534	Tot4	-0.60 (-0.87, -0.21)	< 0.001	0.040
embedding_dim987	Tot4	0.60 (0.28, 0.79)	< 0.001	0.041
embedding_dim878	Tot4	-0.60 (-0.80, -0.26)	0.001	0.041
embedding_dim601	Tot4	0.60 (0.21, 0.84)	0.001	0.041
embedding_dim900	Tot4	0.60 (0.18, 0.86)	0.001	0.041
embedding_dim567	Tot4	0.59 (0.24, 0.83)	0.001	0.042
embedding_dim653	Tot4	0.59 (0.22, 0.82)	0.001	0.042
embedding_dim470	Tot4	0.59 (0.22, 0.81)	0.001	0.042
embedding_dim266	Tot4	0.59 (0.19, 0.82)	0.001	0.044
embedding_dim363	Tot4	0.58 (0.21, 0.83)	0.001	0.044
embedding_dim824	Tot4	0.58 (0.26, 0.79)	0.001	0.044
embedding_dim689	Tot4	0.58 (0.23, 0.81)	0.001	0.044
embedding_dim31	Tot4	-0.58 (-0.82, -0.21)	0.002	0.044
embedding_dim926	Tot4	0.58 (0.18, 0.83)	0.002	0.044
embedding_dim50	Tot4	0.58 (0.19, 0.82)	0.002	0.045
embedding_dim531	Tot4	-0.58 (-0.84, -0.19)	0.002	0.045
embedding_dim749	Tot4	0.57 (0.20, 0.83)	0.002	0.045
embedding_dim1018	Tot4	0.57 (0.20, 0.85)	0.002	0.045
embedding_dim792	Tot4	0.57 (0.21, 0.82)	0.002	0.045
embedding_dim404	Tot4	0.57 (0.27, 0.78)	0.002	0.045
embedding_dim548	Tot4	-0.57 (-0.79, -0.20)	0.002	0.047
embedding_dim865	Tot4	0.57 (0.24, 0.81)	0.002	0.047
embedding_dim716	Tot4	0.57 (0.17, 0.84)	0.002	0.047
embedding_dim907	Tot4	0.57 (0.18, 0.82)	0.002	0.047
embedding_dim593	Tot4	0.56 (0.26, 0.77)	0.002	0.047
embedding_dim853	Tot4	0.56 (0.14, 0.84)	0.002	0.047
embedding_dim66	Tot4	0.56 (0.12, 0.83)	0.002	0.047
embedding_dim620	Tot4	0.56 (0.19, 0.80)	0.002	0.047
embedding_dim528	Tot4	0.56 (0.20, 0.80)	0.002	0.047
embedding_dim999	Tot4	0.56 (0.23, 0.78)	0.002	0.047
embedding_dim886	Tot4	0.56 (0.16, 0.82)	0.002	0.048

Table 5 Elastic Net variable-selection frequency across LOPO-CV folds for the non-embedding feature set. The table reports the top 10 features per MDS-UPDRS target for brevity.

Target	Feature	Selected folds	Selection (%)	Mean β	Mean $\|\beta\|$
Tot1	M10	27/27	100.0	1.302	1.302
Tot1	ACC_day_LIG_bts_1_5_mg	27/27	100.0	-0.681	0.681
Tot1	Light (min)_AT	27/27	100.0	-0.574	0.574
Tot1	ACC_spt_mg_median	27/27	100.0	0.526	0.526

Continued on next page

Target	Feature	Selected folds	Selection (%)	Mean β	Mean $\|\beta\|$
Tot1	ACC_day_IN_unbt_mg	27/27	100.0	0.481	0.481
Tot1	ACC_spt_wake_MOD_mg	27/27	100.0	0.477	0.477
Tot1	MVPA_E5S_B10M80%_T100_ENMO_0-6hr	27/27	100.0	0.431	0.431
Tot1	IV	27/27	100.0	-0.387	0.387
Tot1	L5_ENMO_mg_0-24hr	27/27	100.0	0.344	0.344
Tot1	SleepRegularityIndex1	27/27	100.0	0.276	0.276
Tot2	ACC_spt_wake_IN_mg	27/27	100.0	0.905	0.905
Tot2	ACC_spt_wake_MOD_mg	27/27	100.0	0.743	0.743
Tot2	L5	27/27	100.0	0.576	0.576
Tot2	ACC_spt_mg_median	27/27	100.0	0.557	0.557
Tot2	M10	27/27	100.0	0.549	0.549
Tot2	MVPA_E5S_B10M80%_T100_ENMO_6-12hr	27/27	100.0	-0.484	0.484
Tot2	number_sib_sleepperiod	27/27	100.0	-0.443	0.443
Tot2	number_of_awakenings	27/27	100.0	-0.443	0.443
Tot2	Nblocks_spt_sleep	27/27	100.0	-0.348	0.348
Tot2	MVPA_E5S_B5M80%_T100_ENMO_6-12hr	27/27	100.0	-0.343	0.343
Tot3	ACC_day_MOD_unbt_mg	27/27	100.0	-2.159	2.159
Tot3	RA_AT	27/27	100.0	-2.052	2.052
Tot3	ACC_spt_wake_IN_mg	27/27	100.0	1.586	1.586
Tot3	M5hr_ENMO_mg_0-24hr	27/27	100.0	1.425	1.425
Tot3	Nbouts_day_IN_bts_30	27/27	100.0	-1.263	1.263
Tot3	Nblocks_day_IN_bts_30	27/27	100.0	-1.256	1.256
Tot3	MVPA_E5S_B10M80%_T100_ENMO_6-12hr	27/27	100.0	-1.185	1.185
Tot3	IV1_AT	27/27	100.0	1.059	1.059
Tot3	ACC_spt_mg_median	27/27	100.0	1.050	1.050
Tot3	MVPA_E5S_B5M80%_T100_ENMO_6-12hr	27/27	100.0	-0.908	0.908
Tot4	ACC_spt_wake_IN_mg	27/27	100.0	0.664	0.664
Tot4	dur_day_MOD_unbt_min	27/27	100.0	0.309	0.309
Tot4	ACC_day_IN_bts_10_20_mg	27/27	100.0	0.309	0.309
Tot4	Nblocks_day_MOD_unbt	27/27	100.0	0.258	0.258
Tot4	M10	27/27	100.0	0.224	0.224
Tot4	Nbouts_day_MVPA_bts_1_5	27/27	100.0	0.217	0.217
Tot4	Nblocks_day_MVPA_bts_1_5	27/27	100.0	0.214	0.214
Tot4	MVPA_E5S_T100_ENMO_18-24hr	27/27	100.0	0.198	0.198
Tot4	mean_ENMO_mg_18-24hr	27/27	100.0	0.186	0.186
Tot4	Nblocks_day_LIG_unbt	27/27	100.0	0.173	0.173
Tot_UPDRS	ACC_spt_wake_MOD_mg	27/27	100.0	3.033	3.033
Tot_UPDRS	ACC_spt_wake_IN_mg	27/27	100.0	2.915	2.915

Continued on next page

Target	Feature	Selected folds	Selection (%)	Mean β	Mean $|\beta|$
Tot_UPDRS	RA_AT	27/27	100.0	-2.836	2.836
Tot_UPDRS	M10	27/27	100.0	2.367	2.367
Tot_UPDRS	Nbouts_day_IN_bts_30	27/27	100.0	-2.314	2.314
Tot_UPDRS	Nblocks_day_IN_bts_30	27/27	100.0	-2.304	2.304
Tot_UPDRS	M5hr_ENMO_mg_0-24hr	27/27	100.0	2.281	2.281
Tot_UPDRS	MVPA_E5S_B10M80%_T100_ENMO_6-12hr	27/27	100.0	-2.276	2.276
Tot_UPDRS	ACC_spt_mg_median	27/27	100.0	2.220	2.220
Tot_UPDRS	IV1_AT	27/27	100.0	1.939	1.939

Note. Selection frequency denotes the number and percentage of LOPO-CV folds in which a feature received a non-zero Elastic Net coefficient. Coefficients were estimated after fold-specific standardization using only the training participants. Mean β denotes the average signed coefficient across folds, and mean $|\beta|$ denotes the average absolute coefficient. The table is intended as a descriptive summary of feature-selection stability.

REFERENCES

Adams, J. L., Dinesh, K., Snyder, C. W., Xiong, M., Tarolli, C. G., Sharma, S., et al. (2021). A real-world study of wearable sensors in Parkinson's disease. *npj Parkinson's Disease* 7, 106

Ben-Shlomo, Y., Darweesh, S., Llibre-Guerra, J., Marras, C., San Luciano, M., and Tanner, C. (2024). The epidemiology of Parkinson's disease. *The Lancet* 403, 283–292

Blume, C., Santhi, N., and Schabus, M. (2016). 'nparACT' package for R: A free software tool for the non-parametric analysis of actigraphy data. *MethodsX* doi:10.1016/j.mex.2016.05.006

Bougea, A. (2025). Application of wearable sensors in Parkinson's disease: state of the art. *Journal of Sensor and Actuator Networks* 14, 23

Bremm, R. P., Pavelka, L., Garcia, M. M., Mombaerts, L., Krüger, R., and Hertel, F. (2024). Sensor-based quantification of MDS-UPDRS III subitems in Parkinson's disease using machine learning. *Sensors* 24, 2195

de Graaf, D., de Vries, N. M., van de Zande, T., Schimmel, J. J., Shin, S., Kowahl, N., et al. (2024). Measuring Physical Functioning Using Wearable Sensors in Parkinson Disease and Chronic Obstructive Pulmonary Disease (the Accuracy of Digital Assessment of Performance Trial Study): Protocol for a Prospective Observational Study. *JMIR Research Protocols* 13, e55452

Dias, S. B., Grammatikopoulou, A., Diniz, J. A., Dimitropoulos, K., Grammalidis, N., Zilidou, V., et al. (2020). Innovative Parkinson's disease patients' motor skills assessment: the i-prognosis paradigm. *Frontiers in Computer Science* 2, 20

Farabolini, G., Baldini, N., Pagano, A., Andrenelli, E., Pepa, L., Morone, G., et al. (2025). Continuous movement monitoring at home through wearable devices: A systematic review. *Sensors* 25, 4889

Hssayeni, M. D., Jimenez-Shahed, J., Burack, M. A., and Ghoraani, B. (2021). Ensemble deep model for continuous estimation of Unified Parkinson's Disease Rating Scale III. *Biomedical engineering online* 20, 32

Kanellos, F. S., Tsamis, K. I., Rigas, G., Simos, Y. V., Katsenos, A. P., Kartsakalis, G., et al. (2023). Clinical evaluation in Parkinson's disease: is the golden standard shiny enough? *Sensors* 23, 3807

Mishra, B., Sudheer, P., Rajan, R., Agarwal, A., Srivastava, M. P., Nilima, N., et al. (2024). Bridging the gap between statistical significance and clinical relevance: a systematic review of minimum clinically important difference (mcid) thresholds of scales reported in movement disorders research. *Heliyon* 10
